# Development of Phosphate-Functionalized Magnetic Core–Shell Nanoadsorbent for Rare Earth Element Recovery from LCD Waste

**DOI:** 10.3390/nano16140867

**Published:** 2026-07-15

**Authors:** Javiera Catriñir, José Gaete, Pablo Fuentealba, Gonzalo Montes-Atenas, Fernando Valenzuela, Carlos Basualto

**Affiliations:** 1 Laboratory of Unit Operations and Hydrometallurgy, Faculty of Chemical and Pharmaceutical Sciences, Universidad de Chile, Santiago 8380481, Chile; javiera.catrinir@ug.uchile.cl (J.C.); pfuentealbacastro@ciq.uchile.cl (P.F.); fvalenzu@uchile.cl (F.V.); 2 Research and Development Center in Engineering Sciences (CIDCI), Faculty of Engineering, Science and Technology, Bernardo O’Higgins University, Av. Viel 1497, Santiago 8370993, Chile; jose.gaete.c@ubo.cl; 3 Minerals and Metals Characterisation and Separation Research Group, Department of Mining Engineering, Universidad de Chile, Santiago 8370448, Chile; gmontesa@uchile.cl

**Keywords:** phosphorus functionalized nanoparticles, rare earth recovery, magnetic nanoadsorbent, LCD waste recycling

## Abstract

This work describes the development of a core–shell magnetic nanoadsorbent (Fe_3_O_4_@TiO_2_) designed for the selective recovery of rare earth elements (REEs) from electronic waste. The synthesis involved the co-precipitation of magnetite coated with an anatase-phase TiO_2_ layer, subsequently functionalized with organophosphorus groups using glycolic acid and phosphoric acid. This surface modification, verified via FT-IR spectroscopy and zeta potential analysis, provided the material with a high density of active sites. Adsorption studies with lanthanum revealed that the process follows pseudo-second-order kinetics, reaching equilibrium in only 15 min with a theoretical model-calculated capacity of 19.4 ± 0.8 mg/g at pH 5. The material demonstrated high stability and reusability, maintaining 75% of its adsorption capacity after five cycles with a corresponding H_2_SO_4_ desorption efficiency of 58–60%. Finally, the nanoadsorbent was validated on real LCD screen leachates following an upstream pH 5.0 pre-neutralization and filtration stage designed to remove massive baseline concentrations of iron and copper. Although residual copper and chromium acted as the primary competitors within the remaining complex matrix, the material effectively partitioned REEs (Gd, Y, Ce, Pr, Nd, and Sm) present at ultra-low trace levels (μg/L), demonstrating its potential for urban mining and the circular economy.

## 1. Introduction

The term Waste Electrical and Electronic Equipment (WEEE) refers to discarded technological products that have reached the end of their functional life, a process driven by mechanical failure or technological obsolescence resulting from rapid innovation [1]. Consequently, the integration of new features and surging consumer demand have significantly shortened the lifespan of these devices, resulting in a massive accumulation of electronic waste [2,3]. This surge in WEEE has led to a substantial increase in landfill volume, posing severe environmental risks through the open exposure of hazardous components like heavy metals, which can lead to widespread soil and water contamination [4].

Liquid Crystal Displays (LCDs) and Light-Emitting Diode (LED) screens commonly found in televisions, monitors, and smartphones constitute a major portion of this waste stream. These high-turnover devices’ lifespans have plummeted from eight to two years for large equipment, and from four to one year for mobile phones [2]. Despite their toxicity, these devices contain high concentrations of valuable transition metals and rare earth elements (REEs) [5,6]. According to the 2022 Global E-waste Monitor, 62 million metric tons of e-waste were generated worldwide, including 31 million tons of metal and 17 million tons of plastic, yet only 22.3% was formally collected and recycled through environmentally sound channels [7]. Therefore, optimizing recovery processes is imperative not only to mitigate ecological damage but also to reclaim strategic materials and promote a circular economy.

Current recycling initiatives primarily focus on recovering REEs from the fluorescent lamps found in LCD units—a research trajectory that is increasingly critical for advancing a sustainable electronics industry. REEs are essential across principal applications like permanent magnets, nickel–metal hydride (NiMH) batteries, and fluorescent phosphors [8]. Consequently, these elements constituent concentrated sources of REEs offering substantial research potential despite significant technical complexities [9]. The recovery process from LCD e-waste entails several rigorous preliminary stages: collection, dismantling, and polarizer removal, followed by grinding and pulverization to enable precise qualitative and quantitative sample analysis. Subsequent to these steps, the materials undergo leaching with various mineral acid mixtures under controlled conditions to evaluate elemental recoverability. Once the samples are fully leached, the resulting pregnant leach solutions (PLS) are processed via several separation techniques, including chemical precipitation, ion exchange (IX), solvent extraction (SX), adsorption, and membrane processes. Among these, ion exchange and solvent extraction remain the most prevalent methodologies in current practice.

Solvent extraction (SX) has been extensively utilized for the separation and recovery of metal ions, where metals are favorably partitioned between two immiscible liquid phases—selectively concentrating the target metal in one phase while leaving impurities in the other. SX possesses the dual capability of isolating a metal from a relatively impure solution while simultaneously concentrating it; however, its effectiveness is highly dependent on the specific metal and impurities involved. Numerous extractant families have been developed based on their extraction mechanisms, generally classified into cationic (acidic), anionic (basic), and solvating (neutral) categories [10]. Despite their high specificity, SX technology poses environmental risks and requires significant capital investment for large-scale equipment and chemical inventories. Furthermore, industrial phenomena such as aqueous–organic entrainment, crud formation, and third-phase formation are frequent causes of reduced process efficiency.

One of the most compelling alternatives to SX is the use of adsorption methods onto bed materials like activated charcoal, alumina, silica, and metal oxides. Natural materials such as bentonite, chitosan, and cellulose have also been employed owing to their cost-effectiveness, abundance, and simplicity of use [11,12,13]. However, the primary obstacle to large-scale application is limited specific surface area, which often leads to process inefficiencies. Improving performance requires utilizing the smallest possible particles, as nanoparticulate adsorbent materials (NPs) offer the highest attainable relative surface area. Chemically active nanoparticles typically range from 1 to 10 nm, a scale at which the proportion of surface atoms becomes extraordinarily high. For instance, in a 1 nm diameter particle, approximately 50% of the atoms are surface atoms, a percentage that decreases exponentially as diameter increases. Consequently, these particles exhibit unique physical and chemical properties that closely resemble those of their constituent molecular components [14,15,16,17,18,19]. Nanotechnology is centered on the manipulation of materials at the nanometer scale where materials exhibit emergent properties. The relatively large specific surface area of NPs is comparable to that of porous materials but without associated resistance to mass transport. While extremely small particulate matter increases the surface-to-mass ratio, it introduces a significant challenge: the separation step is often remarkably difficult, typically requiring high-speed centrifugation.

Magnetic nanostructuring has emerged as a transformative phenomenon to address this issue. The magnetic properties of certain adsorbents facilitate the separation of the loaded material from the spent solution—a task otherwise technically demanding as the current cumbersome methods of filtration or centrifugation, among others. The magnetically assisted chemical separation (MACS) method offers a novel approach to isolating species from aqueous media, combining the selectivity of chemical extraction with the ease of magnetic removal [13]. In recent years, magnetic nanoparticles (MNPs)—particularly magnetite-based structures—have garnered interest due to diverse applications in Magnetic Resonance Imaging, targeted drug delivery, and metal ion separation [12]. This is achieved by coating the MNP surface with various functional groups to confer specific adsorption capacities—a process referred to as “functionalization.” In terms of physical characterization, magnetic induction is measured in teslas (T) or gauss (G), while magnetic field strength is measured in amperes per meter (A/m) or oersteds (Oe). To ensure proper retention of these MNPs during processing, a magnetic field of at least 3500 G is required, easily attained using neodymium permanent magnets. Magnetism originates from the movement of negative electric charges and at the atomic level is due to electrons orbiting the nucleus, generating specific magnetic moments determined by orbital and spin quantum numbers. In solid materials, elemental magnets often align to form discrete magnetic units known as “magnetic domains,” the orientation of which defines overall magnetic properties [20]. Materials are classified as paramagnetic, superparamagnetic, diamagnetic, ferromagnetic, antiferromagnetic, or ferrimagnetic based on their response to an external magnetic field (H). Magnetite (Fe_3_O_4_) is a naturally occurring iron oxide with a cubic inverse spinel structure conventionally represented as Fe^3+^[Fe^2+^, Fe^3+^]O_4_ [21].

Superparamagnetism is a phenomenon observed in certain magnetic materials below their critical temperature when the energy to change magnetic moment direction is comparable to thermal energy, leading to random orientation reversals [22]. While bulk magnetite is strongly ferrimagnetic, it transitions to superparamagnetic behavior when crystal size is reduced to the nanometer scale. Chemical co-precipitation from aqueous ferric-ferrous solutions is the most widely utilized synthesis method, where precise pH control is essential to produce superparamagnetic Fe_3_O_4_ and prevent byproducts like goethite or hematite [23].

Functionalization is the methodology employed to coat surfaces with various functional groups through strong adsorption or chemical bonding, serving as a powerful tool in materials science [24]. Functionalized magnetite is particularly attractive due to its high magnetic susceptibility, abundance, and ease of manipulation. MNP surfaces are frequently modified using silicate coupling agents—such as APTES, APTS, and MPTES—or anatase-phase titanium dioxide to achieve a high density of functional end groups, thereby facilitating the subsequent attachment of metals or polymers [25,26,27,28]. In both instances, the formation of a silica or anatase shell yields a refractory protective coating that enhances the chemical stability of the MNP core [29,30,31]. Over the past several years, numerous magnetic adsorbents have been developed for metal recovery and contaminant removal, such as magnetic carrageenan/silica nanoadsorbents or green hybrid magnetic particles within a chitosan matrix [32,33]. The Fe_3_O_4_ core provides high magnetic saturation capacity facilitating rapid separation, while the silica or anatase shell provides essential chemical stability in acidic solutions. Functionalization with alkylphosphorus acids enables these materials to serve as adsorbents that emulate the selectivity of successful solvent extractants.

Furthermore, employing an adsorption-based approach rather than organic solvents mitigates the risks of toxicity and flammability inherent in traditional solvent extraction (SX), while preventing industrial limitations such as crud formation and entrainment.

To clearly contextualize the scientific contribution of this study, it is essential to contrast the proposed nanomaterial with existing benchmarks in the literature. While magnetic core–shell nanoadsorbents frequently utilize silica (SiO_2_) shells as basic protective barriers, recent research has increasingly explored anatase-phase titanium dioxide (TiO_2_) shells as highly chemical-resistant, refractory platforms capable of outstanding structural preservation under the highly corrosive, strongly acidic conditions required for processing electronic waste. Although analogous Fe_3_O_4_@TiO_2_ core–shell structures have been previously reported for rare earth element (REE) recovery, they typically rely on the direct grafting of single-acid ligands or traditional organophosphorus extractants, which often suffer from prolonged mass transport limitations requiring hours to reach full equilibrium. In contrast, our design focuses on the specific integration of an anatase-phase TiO_2_ shell with a GA/H_3_PO_4_ dual functionalization pathway. Operationally, this tailored combination overcomes traditional diffusion barriers, achieving exceptionally rapid adsorption kinetics that reach complete equilibrium in only 15 min while providing a highly robust framework engineered for competitive multi-element extraction. Finally, the practical merit of this nanoadsorbent goes beyond idealized laboratory models; it displays a robust multi-cycle reuse performance (retaining 75% of adsorption capacity over 5 cycles) and its operational selective capacity is thoroughly validated using real, complex matrices derived from multi-elemental LCD screen leachates.

## 2. Objectives

- Synthesize and characterize a tailored core–shell magnetic nanomaterial consisting of a magnetite core shielded by a chemically resistant anatase-phase titanium dioxide layer, optimized for acidic hydrometallurgical operations.
- Functionalize the surface: Incorporate organophosphorus groups using glycolic and phosphoric acids to provide the material with specific active sites.
- Determine kinetic and equilibrium parameters: Identify the mechanisms governing the adsorption process and determine the maximum loading capacity, using La^3+^ as a model ion.
- Evaluate nanoadsorbent performance: Test its capacity to recover rare earth elements (REEs) from acid leaching solutions derived from waste electrical and electronic equipment (WEEE).
- Validate stability and reusability: Assess the number of adsorption–desorption cycles and regeneration steps with sulfuric acid (H_2_SO_4_) that the material can undergo before experiencing a significant loss in efficiency.

## 3. Materials and Methods

### 3.1. Materials and Reagents

The following inorganic reagents were used as received from the supplier without further purification: hydrochloric acid (HCl, 37%), sulfuric acid (H_2_SO_4_, 95–97%), nitric acid (HNO_3_, 65%), ortho-phosphoric acid (H_3_PO_4_, 85%), diammonium phosphate ((NH_4_)_2_HPO_4_), sodium hydroxide (NaOH), iron(III) chloride hexahydrate (FeCl_3_·6H_2_O), iron(II) chloride tetrahydrate (FeCl_2_·4H_2_O), and ammonium hydroxide (NH_4_OH, 25%), all purchased from Merck, Darmstadt, Germany, (analytical grade, p.a.). Titanium(IV) isopropoxide (C_12_H_28_O_4_Ti, 98%), lanthanum(III) nitrate hexahydrate (La(NO_3_)_3_·6H_2_O), cerium(III) nitrate hexahydrate (Ce(NO_3_)_3_·6H_2_O), praseodymium(III) nitrate hexahydrate (Pr(NO_3_)_3_·6H_2_O), neodymium(III) oxide (Nd_2_O_3_), samarium(III) oxide (Sm_2_O_3_), and europium(III) nitrate pentahydrate (Eu(NO_3_)_3_·5H_2_O) were obtained from Sigma-Aldrich, St. Louis, MO, USA, (analytical grade, p.a.).

Similarly, the organic reagents were used as received from the suppliers without further purification. Glycolic acid (99% purity, C_2_H_4_O_3_) and Arsenazo III (C_22_H_18_As_2_N_4_O_14_S_2_) were purchased from Sigma-Aldrich (analytical grade, p.a.). Absolute ethanol (C_2_H_6_O), Span 80 (sorbitan monooleate, C_24_H_44_O_6_), and urea (CH_4_N_2_O) were obtained from Merck (analytical grade, p.a.). Technical grade hexane (C_6_H_14_) was supplied by Oregon Chem Group, Santiago, Chile.

### 3.2. Characterization Techniques

- The zeta potential and hydrodynamic size (DLS) of the functionalized nanoparticles were measured using a Zetasizer Nano ZS, Malvern Instruments, Malvern, UK. For zeta potential analysis, samples were dispersed in distilled water with the pH adjusted between 2 and 10 using dilute 0.1 M HCl and 0.1 M NaOH solutions. For DLS measurements, 5 mg of sample was dispersed in 100 mL of distilled water adjusted to pH 5. In both cases, suspensions were prepared in test tubes and homogenized via ultrasonication for 30 min prior to measurement.
- Fourier-Transform Infrared Spectroscopy (FT-IR)
  FTIR Spectra were recorded in the 4000–500 cm^−1^ range using an Attenuated Total Reflectance Accessory with an ZnSe crystal, coupled to an JASCO 4100 spectrophotometer, Tokyo, Japan.
- Transmission Electron Microscopy (TEM)
  Morphological characterization was performed using a Thermo Fisher Talos F200C TEM, Waltham, MA, USA, operated at 200 kV. The instrument is equipped with an X-FEG field emission source and a Ceta 16M (4k × 4k) CMOS camera, providing a maximum resolution of 0.18 nm in both TEM and STEM modes. Samples were prepared by dispersing the powder in ethanol and depositing a droplet onto a copper grid.
- Magnetic Properties
  Magnetic data were collected using a Quantum Design Inc., San Diego, CA, USA equipment, model PPMS Dynacool 2016 by obtaining hysteresis loops at room temperature. Magnetization was measured in emu/g against an applied external magnetic field ranging from −10,000 to 10,000 Oe. Approximately 10 mg of dry sample was used for each measurement to ensure accurate mass-normalized results.
- X-ray Diffraction (XRD)
  The crystalline structure was analyzed using a Bruker, Karlsruhe, Germany, D8 Advance powder diffractometer in Bragg–Brentano geometry. The system utilized CuKα = 1.5406 Å operated at 40 kV and 30 mA, with a nickel filter to remove Kβ radiation. Patterns were recorded over a 2θ = 10–80° using a LynxEye linear detector and variable optics adjusted for each measurement.
- ICP-MS
  Rare earth element (REE) quantifications were performed using an iCAP RQ inductively coupled plasma mass spectrometer (ICP-MS) from Thermo Scientific, Waltham, MA, USA. The instrument was equipped with a 4DX autosampler (Elemental Scientific) to ensure precise sample introduction. All measurements were conducted under optimized operating conditions to maximize sensitivity and minimize oxide interferences during the analysis.

### 3.3. Methods

#### 3.3.1. Synthesis Phosphate-Functionalized Magnetic Core–Shell Nanoparticles

Step 1: Synthesis of Magnetite Core (MNPs)

Magnetite nanoparticles were synthesized via chemical co-precipitation. Fe(III) chloride hexahydrate (2.7 g) and Fe(II) chloride tetrahydrate (1.0 g) (2:1 molar ratio) were dissolved in 100 mL of degassed deionized water. The solution was adjusted to pH 2.0 using 0.1 M HCl to prevent premature iron precipitation. Under mechanical stirring at 300 rpm and a constant temperature of 70 °C, 15 mL of ammonium hydroxide (NH_4_OH, 25% *w*/*w*) was added dropwise at a controlled rate of approximately 1.5 mL/min using a peristaltic pump. The reaction was maintained under these conditions for 30 min. The resulting black precipitate was isolated via magnetic decantation (10 min exposure to a 3500 G neodymium magnet) and washed three times with 50 mL of deionized water per cycle to remove residual reagents, yielding approximately 1.1 g of wet MNPs [34].

Step 2: Titanium Dioxide Coating (MNP@TiO_2_)

To form the core–shell structure, the freshly prepared wet MNPs (1.1 g) were dispersed in 100 mL of deionized water inside a three-necked flask via ultrasonication for 10 min, followed by continuous mechanical stirring at 300 rpm. Separately, a titanium precursor solution was prepared by dissolving 2.5 mL of titanium(IV) tetraisopropoxide (TTIP, 98%) in 20 mL of absolute ethanol (yielding a TTIP concentration of approximately 0.42 M). This TTIP solution was added dropwise to the MNP suspension at a controlled volumetric rate of 0.5 mL/min under vigorous mechanical stirring at 70 °C. After complete addition, stirring was maintained for 20 min to finish the coating process. The intermediate MNP@TiO_2_ nanoparticles were isolated through magnetic decantation (15 min), washed twice with 40 mL of absolute ethanol to remove unreacted TTIP, and three times with 50 mL of deionized water. After drying at 80 °C for 6 h, the dry mass yield of MNP@TiO_2_ was 1.42 g (based on the starting iron cores).

Step 3: Surface Functionalization (MNP@TiO_2_-GA-H_3_PO_4_)

The chemical modification was executed in two successive stages: Glycolic Acid Activation: A total of 1.0 g of dry MNP@TiO_2_ was suspended in 200 mL of an aqueous glycolic acid solution (0.1 M, adjusted to pH 3.0 using 0.1 M NaOH). The mixture was stirred at 300 rpm and ultrasonicated simultaneously in a water bath at 70 °C for 3 h to achieve primary alcohol activation (MNP@TiO_2_-GA). The particles were isolated magnetically and washed three times with 50 mL of deionized water. Phosphorylation Step: The wet MNP@TiO_2_-GA intermediate was immediately transferred and dispersed into an aqueous phosphorylation mixture containing 0.15 mmol of ortho-phosphoric acid (H_3_PO_4_, 85%), 0.2 mmol of diammonium hydrogen phosphate ((NH_4_)_2_HPO_4_), and 1.0 mmol of urea dissolved in 150 mL of deionized water. The reaction was conducted in an ultrasonication bath under identical conditions (70 °C, 300 rpm mechanical stirring) for 3 h. Finally, the synthesized MNP@TiO_2_-GA-H_3_PO_4_ nanoparticles were isolated via magnetic decantation (15 min) [35]. The recovered material was dried at 150 °C for 2 h to ensure stable chemical bonding and subsequently washed five times with 60 mL of deionized water to eliminate non-bonded phosphate species and reaction residues. After final drying at 60 °C for 12 h under vacuum, the final mass yield of the functionalized nanoadsorbent was 1.18 g (calculated relative to the 1.0 g of MNP@TiO_2_ precursor input).

#### 3.3.2. Adsorption Procedures

Adsorption studies were conducted to evaluate the influence of operational parameters on the loading capacity (q) and removal efficiency of the synthesized MNPs. To assess the effect of pH, 100 mg of adsorbent was contacted with 100 mL of 100 mg/L La^3+^ feed solutions with initial pH values ranging from 2 to 6. Adsorbent dosage studies were performed by varying the mass-to-volume ratio between 0.5 and 2 g/L at pH 5. Kinetic experiments involved contacting 100 mg of adsorbent with 100 mL of a 100 mg/L La^3+^ solution at pH 5, with samples collected at time intervals ranging from 3 to 25 min. Equilibrium studies followed a similar procedure but utilized varying initial La^3+^ concentrations between 30 and 100 mg/L until equilibrium was reached. All adsorption tests were carried out at room temperature under mechanical stirring at 300 rpm, and La^3+^ concentrations were quantified using the Arsenazo III method. For desorption and reusability assays, metal-loaded MNPs were recovered, dried, and contacted with eluent solutions of H_2_SO_4_, HNO_3_, or HCl (0.01 and 0.1 M). Regeneration cycles were performed by treating the loaded material with 0.1 M H_2_SO_4_ for 15 min, followed by washing and drying before reintroduction into subsequent adsorption cycles to evaluate long-term stability and efficiency. All initial adsorption experiments, including kinetic, pH effects, dosage, and equilibrium studies, were performed in independent triplicates (n = 3) to ensure reproducibility. Quantitative results and key adsorption outputs throughout the manuscript are reported as the mean ± standard deviation (mean ± SD).

The magnetic separation performance of the adsorbent was evaluated under the same experimental conditions as the adsorption tests. Following each experiment, the material was recovered using an external neodymium magnet, allowing the separation time, recovered mass, and mass loss to be recorded across five consecutive cycles.

#### 3.3.3. Analysis of Rare Earth Elements

The concentration of lanthanum (La^3+^) ions in aqueous solutions was determined using the Arsenazo III colorimetric method [36]. This technique is based on the formation of a highly colored La^3+^-Arsenazo III complex, which exhibits a strong absorption band with a maximum wavelength (λ_max_) at 660 nm. Although Arsenazo III is a versatile reagent for the determination of various rare earth elements, in this study, it was employed specifically to quantify the La^3+^ content. The analytical procedure involved the preparation of three primary solutions: A 1 M HCOOH/HCOO^−^ buffer solution adjusted to pH 3.5. A 5 mM Arsenazo III stock solution. A La^3+^ standard solution for the construction of the calibration curve. For the measurements, 3 mL of the buffer solution, 100 µL of the Arsenazo III solution, and 100 µL of the sample were added to a polystyrene cuvette. The cuvette was then sealed and agitated until a stable color developed. The absorbance was subsequently recorded using a UV-Vis spectrophotometer, Jenway Ltd, Stone, UK. 

Rare earth element (REE) concentrations were determined by ICP-MS using an external multi-element calibration curve with standard concentrations of 0.05, 0.1, 0.5, 1, 5, 10, 50, and 100 ppb. This approach ensured high linearity and precision across the entire analytical range. 

## 4. Results

The findings of this study are organized into four sections. The first characterizes the MNP@TiO_2_ core–shell nanoparticles using zeta potential analysis, Fourier-transform infrared spectroscopy (FT-IR), transmission electron microscopy (TEM), vibrating-sample magnetometry (VSM), and X-ray diffraction (XRD). Collectively, these techniques confirm the successful formation of the intended coating. Second, the surface functionalization of the proposed MNP@TiO_2_-GA-H_3_PO_4_ magnetic adsorbent is addressed, where the incorporation of organophosphorus groups is verified through further zeta potential, FT-IR, TEM, and VSM analyses. Third, the adsorption of lanthanum(III) onto the adsorbent is detailed, evaluating the influence of key operational parameters such as pH, dosage, and contact time. Furthermore, the material’s capacity was assessed against other rare earth elements, alongside desorption and regeneration cycles to establish its long-term stability and multi-cycle efficiency. Finally, a morphological and elemental characterization of end-of-life LCD screens is presented to confirm the presence of rare earth elements. These devices underwent controlled acid leaching to generate leachates containing these ions, which were subsequently subjected to adsorption experiments to evaluate the adsorbent’s performance in complex real-world matrices.

### 4.1. Magnetite@Titanium Dioxide Core–Shell Nanoparticles (MNP@TiO_2_)

MNP@TiO_2_ core–shell nanoparticles were obtained through chemical co-precipitation; subsequently, the titanium dioxide coating was applied according to the procedure described in the methodology. The purpose of this stage was to form a homogeneous TiO_2_ layer on the magnetite surface to enhance its chemical resistance in acidic media and provide a suitable platform for subsequent functionalization.

**Figure 1:**
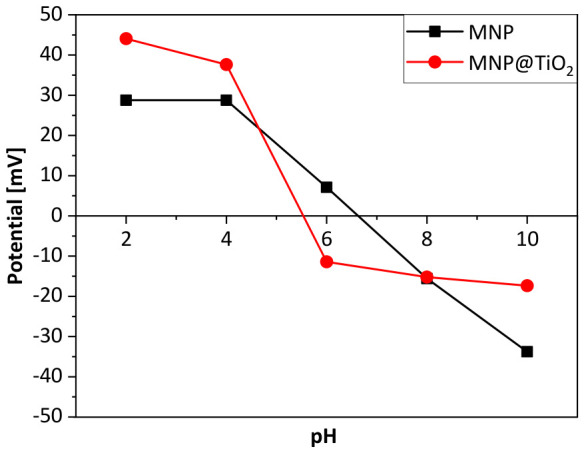
Zeta potential of MNP and MNP@TiO_2_ as a function of pH.

The zeta potential was measured as a function of pH to confirm the surface modification of the magnetite nanoparticles with a TiO_2_ coating. The results for the precursor MNPs and the resulting MNP@TiO_2_ are presented in Figure 1.

As illustrated in Figure 1, the zeta potential curve for MNP@TiO_2_ exhibits a shift compared to the uncoated MNP reference. A decrease in the point of zero charge (PZC) from pH 6.6 to 5.5 was observed upon coating, which verifies the successful modification of the surface. Furthermore, at pH values below the PZC, the nanoparticle surface is positively charged, whereas at higher pH levels, it acquires a negative charge. This behavior allows for the control of surface interactions by promoting or inhibiting electrostatic attraction and repulsion. Therefore, understanding these properties is vital when designing functionalization strategies.

**Figure 2:**
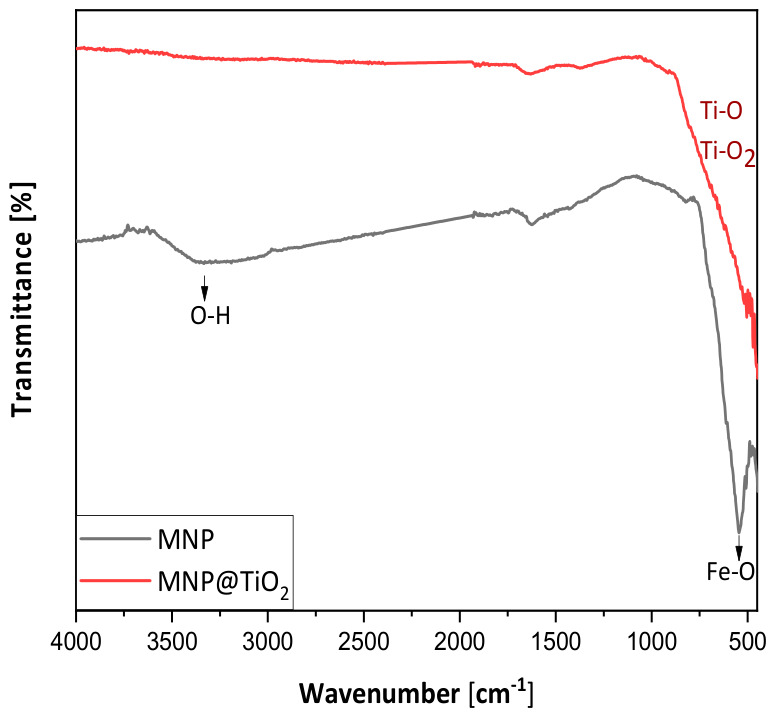
FT-IR spectra of MNP and MNP@TiO_2_.

FT-IR spectroscopy was employed to verify the TiO_2_ coating on the nanoadsorbents. The spectra for the precursor MNPs and the MNP@TiO_2_ samples (Figure 2) were analyzed to identify characteristic absorption bands corresponding to Ti–O bonds.

A band at 3300 cm^−1^ in the MNP spectrum is attributed to O–H stretching vibrations, which indicates the presence of surface hydroxyls or residual water. The peak at 550 cm^−1^ corresponds to the characteristic Fe–O stretching mode [37]. On the other hand, the MNP@TiO_2_ spectrum shows a prominent band in the 500–800 cm^−1^ range, assigned to Ti–O and Ti–O–Ti stretching vibrations. These features confirm the successful deposition of the TiO_2_ layer onto the nanoparticles [38,39].

To characterize the morphology and verify the formation of the TiO_2_ shell on the magnetite nanoparticles, TEM analysis was performed on both MNP and MNP@TiO_2_ samples. This approach enabled a comparative study of the nanoparticle structure and dimensions before and after the coating process. For the statistical particle size distribution, a total of N = 105 nanoparticles were manually measured from the TEM micrographs using image analysis software, ImageJ NIH, Bethesda, MD, USA.

**Figure 3:**
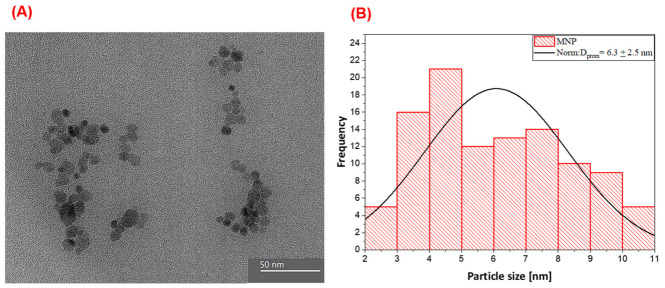
(**A**) TEM micrograph of MNPs; (**B**) particle size distribution of MNPs (N = 105).

The TEM image and corresponding size distribution histogram for the magnetite nanoparticles are shown in Figure 3A and Figure 3B, respectively. The particles display a mainly spherical shape with a unimodal distribution primarily ranging from 2 to 11 nm. The average particle size was determined to be 6.3 ± 2.5 nm, aligning with reported data for magnetite synthesized through the chemical co-precipitation method [40,41,42].

**Figure 4:**
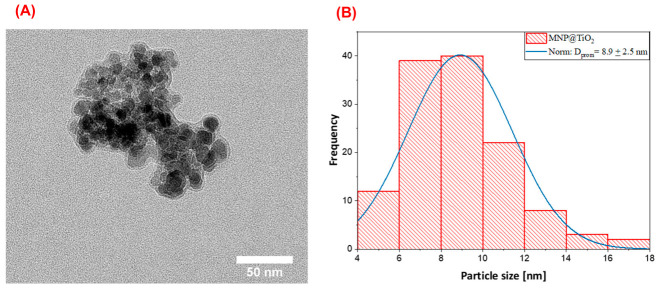
(**A**) TEM micrograph; (**B**) particle size distribution of MNP@TiO_2_ (N = 105).

As observed in Figure 4, the MNP@TiO_2_ nanoparticles retain their spherical shape, displaying a unimodal size distribution primarily between 4 and 18 nm. Comparing the histograms from Figure 3B and Figure 4B indicates a clear increase in average particle diameter after the coating process. This growth confirms the deposition of a TiO_2_ shell, with a calculated thickness of 2.4 ± 0.8 nm, onto the magnetite cores.

**Figure 5:**
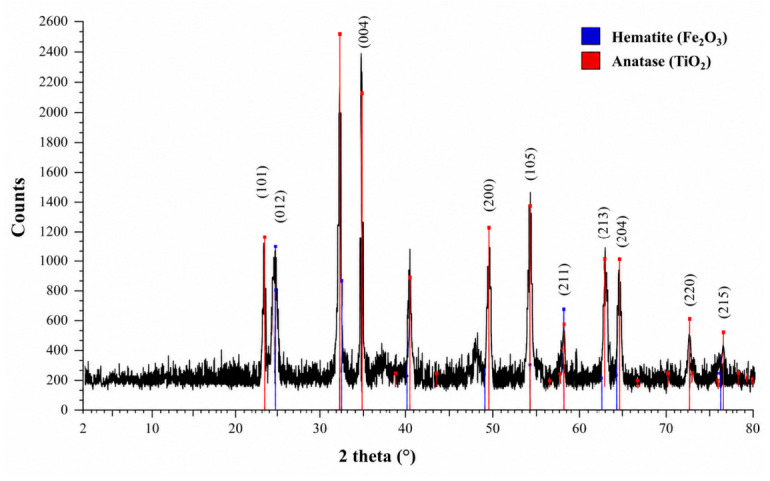
X-ray diffraction (XRD) pattern of MNP@TiO_2_.

X-ray diffraction was employed to characterize the TiO_2_ coating on the nanoparticles. The analysis aimed to identify the TiO_2_ crystalline phases and confirm the presence of anatase within the composite material. The XRD patterns are shown in Figure 5.

The XRD pattern in Figure 5 confirms the successful synthesis of the composite core–shell structure, with the dominant diffraction peaks corresponding to the planes of anatase TiO_2_ (JCPDS 21-1272). Minor signals at 2θ = 25.5° indicate the presence of hematite (α-Fe_2_O_3_), which points to a partial surface oxidation of the iron oxide core during the high-temperature processing steps [43]. Regarding the magnetic core phase, a cautious structural analysis is required. While explicit, standalone reflection peaks strictly indexable to bulk magnetite (Fe_3_O_4_) are not prominently resolved, this structural feature should not be interpreted as a total absence of the magnetite phase. Instead, this phenomenon is attributed to a combination of physical factors typical of core–shell nanomaterials. First, the ultra-small dimension of the core crystallites (6.3 ± 2.5 nm according to TEM) induces severe peak broadening via the Scherrer effect, significantly lowering peak intensities into the background signal. Second, the main characteristic reflection of the inverse spinel magnetite structure—specifically the (311) plane expected at 2θ approx 35.4°—suffers from severe peak overlap with the highly intense reflections of the surrounding anatase shell in the 33–37° region. Lastly, the relatively thick, highly crystalline TiO2 shell layers (2.4 ± 0.8 nm) exert a strong X-ray attenuation and shielding effect over the small inner iron core. Therefore, the persistence of an underlying, superparamagnetic iron oxide core (Fe_3_O_4_) is structurally shielded in the diffractogram, but its definitive presence is unambiguously corroborated by the clear room-temperature superparamagnetic response and macroscopic magnetic collectability confirmed in the subsequent VSM analysis.

**Figure 6:**
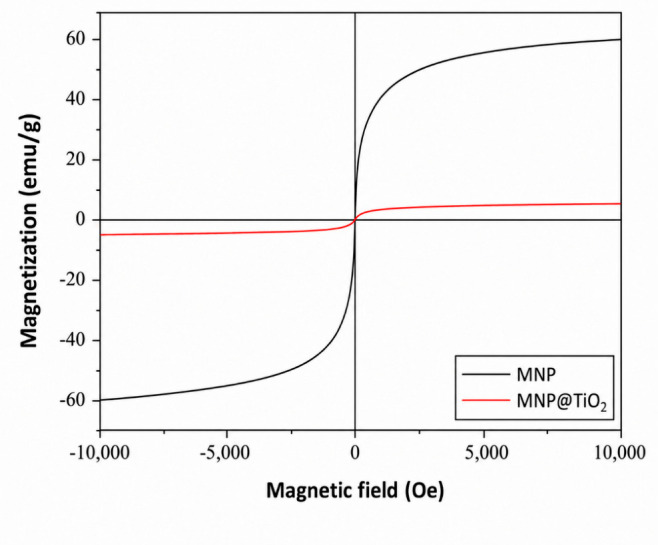
Magnetic hysteresis loops of MNPs and MNP@TiO_2_.

The magnetic properties of MNP and MNP@TiO_2_ were characterized via vibrating-sample magnetometry (VSM). From the resulting hysteresis loops (Figure 6), key parameters such as saturation magnetization (M_s_) and remanent magnetization (M_r_) were determined to assess the impact of surface coating on magnetic performance. These characteristic values are summarized in Table 1.

The MNP sample showed a high M_s_ of 60 emu/g with typical superparamagnetic behavior. Upon coating (MNP@TiO_2_), the M_s_ dropped to 4.7 emu/g due to the shielding effect of the diamagnetic TiO_2_ shell. Nevertheless, the nanoparticles remained superparamagnetic and magnetically recoverable using an external field, though with reduced sensitivity. This behavior aligns with previously reported Fe_3_O_4_@TiO_2_ core–shell systems [23,39].

Collectively, these findings confirm the successful formation of the anatase TiO_2_ coating. Despite the coating, the nanoparticles retain sufficient saturation magnetization for magnetic recovery via an external field. The presence of anatase as the dominant crystalline phase provides a well-defined surface, essential for subsequent functionalization.

### 4.2. Functionalization of MNP@TiO_2_ with GA-H_3_PO_4_

According to literature reports, organophosphorus compounds have demonstrated high affinity for lanthanide ions in solvent extraction processes; this makes them suitable candidates for surface modification of adsorbents intended for the recovery of rare earth elements [41,42].

**Figure 7:**
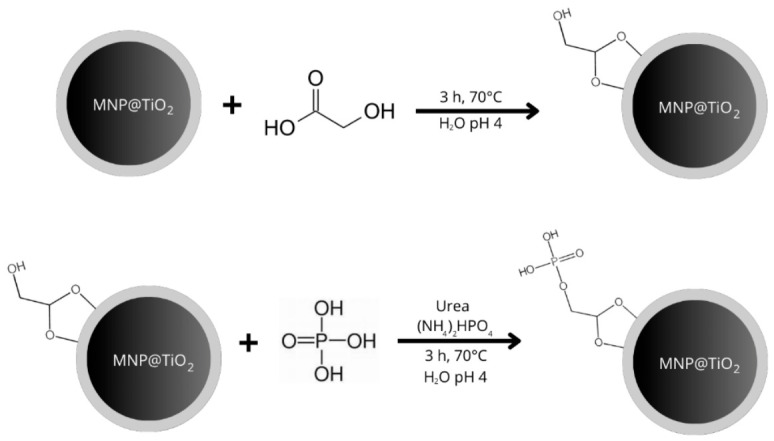
Functionalization of MNP@TiO_2_ with phosphoric acid.

The synthesis of MNP@TiO_2_-GA-H_3_PO_4_ was performed via a two-step route. In the first step, glycolic acid was incorporated onto the surface of the MNP@TiO_2_ through a controlled chemical reaction (Figure 7), yielding MNP@TiO_2_-GA. This step introduced primary alcohol groups onto the surface of the nanomaterial, which subsequently enabled phosphorylation with phosphoric acid in the presence of urea, generating the final MNP@TiO_2_-GA-H_3_PO_4_ material.

**Figure 8:**
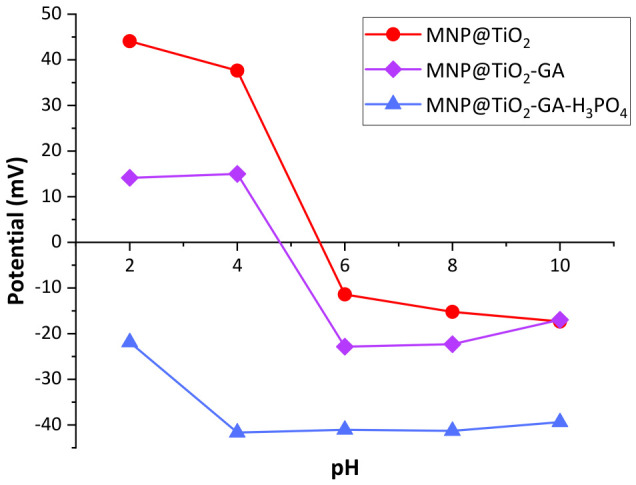
Zeta potential curves of MNP@TiO_2_, MNP@TiO_2_-GA, and MNP@TiO_2_-GA-H_3_PO_4_.

The zeta potential measurements as a function of pH for the MNP@TiO_2_-GA-H_3_PO_4_ adsorbent are presented in Figure 8.

Figure 8 illustrates the PZC behavior of the products obtained at both stages of the MNP@TiO_2_-GA-H_3_PO_4_ synthesis route. While the MNP@TiO_2_ precursor exhibits a PZC at pH 5.5, the product from the first stage shows a value of 4.8. Likewise, the second stage yields negative surface charge values across the entire pH range studied. This phenomenon would evidence a high density of phosphate groups on the surface. It should be noted that phosphoric acid is a medium-strength acid with a pKa value of 2.15 for its first dissociation; therefore, the nanoparticles functionalized with this acid are expected to exhibit a negative charge across the entire pH range studied.

**Figure 9:**
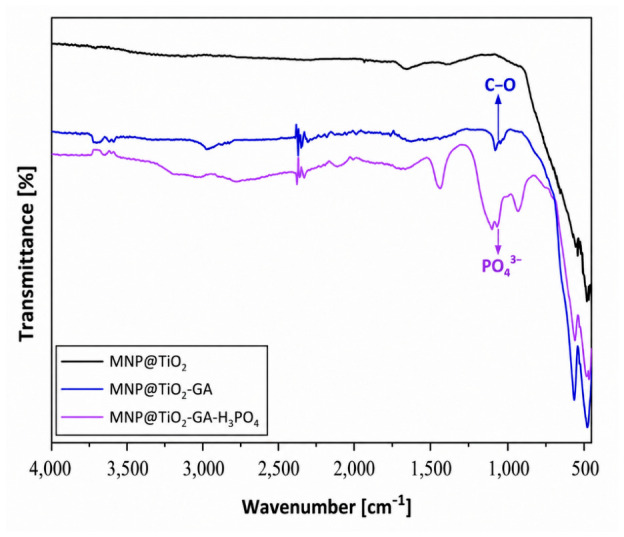
FT-IR spectra of MNP@TiO_2_, MNP@TiO_2_-GA, and MNP@TiO_2_-GA-H_3_PO_4_.

The FT-IR spectra of the MNP@TiO_2_-GA-H_3_PO_4_ sample and its precursors, MNP@TiO_2_ and MNP@TiO_2_-GA, are presented in Figure 9. This analysis allowed for the identification of bands associated with the incorporated functional groups and provided evidence of the structural modifications introduced onto the material’s surface.

In Figure 9, the spectrum of MNP@TiO_2_-GA exhibits a band around 1100 cm^−1^ attributed to the C—O bond vibrations of the acetal group formed during the reaction with glycolic acid. Meanwhile, the MNP@TiO_2_-GA-H_3_PO_4_ spectrum shows a band at approximately 1000 cm^−1^, corresponding to the vibration of the phosphate group (PO_4_^3−^) [44]. The presence of these signals confirms the incorporation of the proposed organic groups onto the surface of the functionalized adsorbents, validating the effectiveness of the required surface modification.

**Figure 10:**
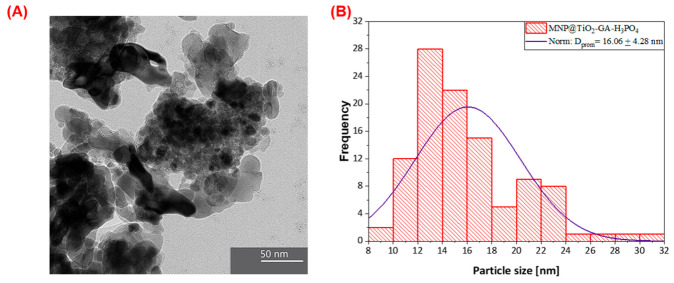
(**A**) TEM image of MNP@TiO_2_-GA-H_3_PO_4_ and (**B**) frequency histogram of the particle diameters (N = 105).

Figure 10 shows TEM micrograph of the functionalized MNP@TiO_2_-GA-H_3_PO_4_, which confirm the material’s morphology and average particle size.

Figure 10A shows that the nanoparticles maintain a predominantly spherical morphology, albeit with some degree of agglomeration. The histogram (Figure 10B), which was also fitted using a normal distribution model, yields an average diameter of 16.1 ± 4.3 nm.

**Figure 11:**
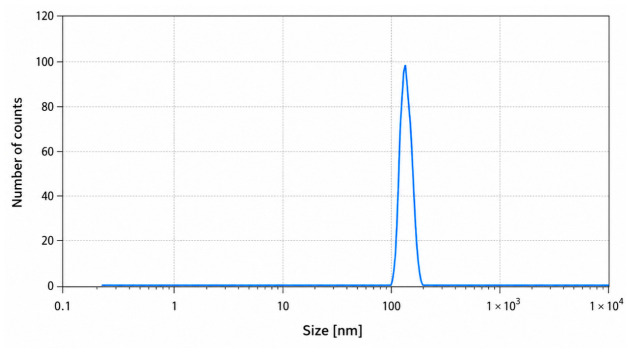
DLS curve of MNP@TiO_2_-GA-H_3_PO_4_.

Dynamic Light Scattering (DLS) was employed to determine the particle size distribution of the materials in the dispersed phase. In this method, a light beam is incident upon the nanoparticle suspension, and the fluctuations in the scattered light intensity are recorded over short time intervals. Smaller nanoparticles generate faster signal fluctuations, whereas larger particles produce slower variations. Measurements were performed for MNP@TiO_2_-GA-H_3_PO_4_ by dispersing 5 mg of the adsorbent nanomaterial in 100 mL of distilled water adjusted to pH 5. The resulting distribution curve is presented in Figure 11.

Given that individual nanoparticles exhibit an average diameter of 16.1 nm while the agglomerates measure 125.6 nm, it can be estimated that each cluster consists of a small fraction of approximately 8 to 10 primary units. Although such aggregation typically compromises the effective specific surface area and introduces mass transfer resistance, the exceptionally rapid adsorption kinetics (reaching full equilibrium in only 15 min) imply that these clusters maintain a highly loose, open porous structure in the aqueous phase. This structural configuration minimizes internal diffusion barriers, preventing severe restriction of intraparticle mass transport to the grafted active phosphate receptors. Consequently, while this aggregation does not enhance internal mass transfer, it acts as a significant operational asset from an engineering standpoint; the larger hydrodynamic size of the multi-core clusters substantially amplifies their collective magnetic response, thereby enhancing macroscopic separation velocity and process scalability without critically sacrificing kinetic performance [45]. Dynamic Light Scattering (DLS) analysis recorded a Polydispersity Index (PDI) of 1.0, confirming a highly polydisperse colloidal suspension.

The effect of surface functionalization on the magnetic properties was evaluated. For this purpose, the MNP@TiO_2_-GA-H_3_PO_4_ sample was analyzed using vibrating sample magnetometry (VSM). The results obtained were compared with the MNP@TiO_2_ sample used as a reference.

**Figure 12:**
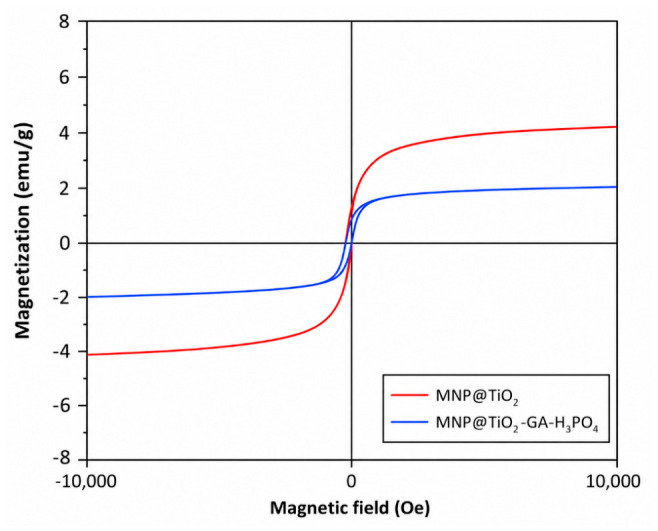
Magnetization curves of MNP@TiO_2_ and MNP@TiO_2_-GA-H_3_PO_4_.

Figure 12 and Table 2 illustrate a decrease in saturation magnetization (Ms) upon the incorporation of organophosphorus groups onto the MNP@TiO_2_ surface. The MNP@TiO_2_ sample exhibited an Ms of 4.7 emu/g, whereas that of the functionalized MNP@TiO_2_-GA-H_3_PO_4_ decreased to 1.9 emu/g. This reduction is attributed to the presence of non-magnetic layers on the material’s surface, which increase the total magnetic dead mass without contributing to the net magnetic moment, thereby diluting the overall magnetic response [43]. Despite the reduction in M_s_, the functionalized nanoparticles maintain their superparamagnetic behavior, as evidenced by the absence of significant remanent magnetization. Furthermore, the M_r_/M_s_ ratio remained below 0.1 in all cases, confirming that the nanoparticles retain their superparamagnetic character. This property is essential for efficient magnetic separation in liquid media; thus, despite the relatively low M_s_ values, magnetic recovery remains feasible.

To evaluate operational viability, magnetic separation experiments were conducted across five consecutive cycles under identical adsorption conditions. As compiled in Table 3, the magnetic recovery efficiency consistently remained between 91.5% and 99.7%, confirming robust solid–liquid separation despite the material’s relatively low saturation magnetization. The observed mass losses were minor and primarily driven by physical material transfer during the washing and recovery steps, rather than any degradation of underlying magnetic responsiveness.

This high separation performance is governed by a delicate balance between colloidal stability and active magnetic responsiveness. At the operational pH of 5.0, zeta potential measurements reveal a highly negative surface charge of approximately −33 mV. This substantial electrostatic barrier provides sufficient repulsive force to prevent uncontrolled, irreversible macroscopic coagulation, maintaining a workable and scalable fluidic suspension until active recovery is initiated. Once exposed to an external magnetic field, the suspension’s cluster-size heterogeneity triggers a cooperative “magnetic sweeping” mechanism. The larger multi-core aggregates migrate swiftly due to their high cumulative magnetic susceptibility, physically entangling and sweeping down the smaller co-dispersed cluster fractions. This progressive magnetic aggregation and enhanced settling behavior directly accounts for the remarkable reduction in suspension clearing times, which dropped from 2.0 to 0.6 min over successive handling cycles.

Based on the characterization results presented for the MNPs, it can be established that the magnetite core provides the requisite magnetic properties to facilitate their separation from treated aqueous solutions. Furthermore, the TiO_2_ coating layer protects the nanoparticles from the acidity of the REE-containing solutions, while the surface functionalization with glycolic and phosphoric acids imparts specific adsorption properties toward these target elements.

### 4.3. Adsorption Experiments

The adsorption effectiveness for lanthanide ions onto synthesized nanoparticles was expressed as the loading capacity (*q*), as defined by Equation (1).

(1) $$q=\frac{\left(C_{i}-C_{e}\right)\cdot V}{M}\left[\frac{{mg}_{M^{3+}}}{g_{MNP}}\right]$$

In this equation, *q* represents the loading capacity (mg_M3+_/g_MNP_), V is the volume of the feed solution (L), *C_i_* and *C_e_* are the initial and equilibrium concentrations of the lanthanide ions (mg/L), respectively, and M is the mass (g) of the adsorbent used.

Additionally, the efficiency of the adsorption process was evaluated using the adsorption percentage (A%), which was calculated from the initial and equilibrium concentrations of the lanthanide ions according to Equation (2).

(2) $$A\%=\frac{\left(C_{i}-C_{e}\right)}{C_{i}}\cdot 100$$

where *C_i_* and *C_e_* correspond to the initial and equilibrium concentrations (mg/L), respectively. This parameter allows for the evaluation of the fraction of ions removed from the aqueous solution.

All initial adsorption experiments, including kinetic and equilibrium studies, were conducted using lanthanum(III) as a representative element of the rare earth series due to its similar chemical behavior.

#### 4.3.1. Effect of pH on Lanthanum(III) Adsorption

**Figure 13:**
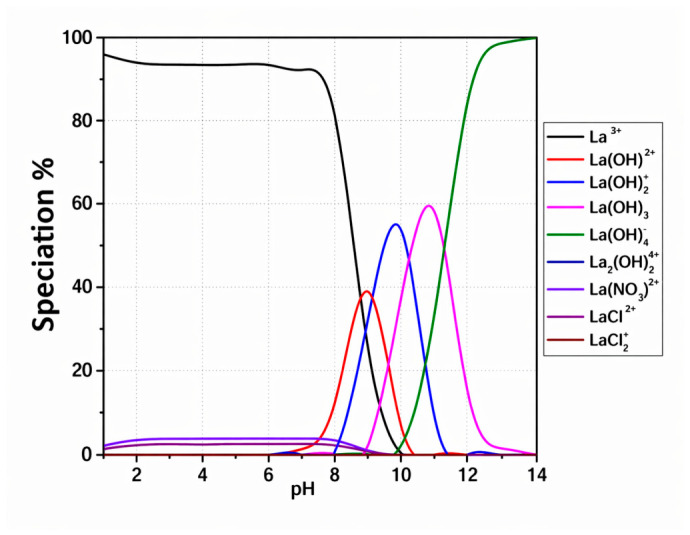
Speciation diagram of La(III) in the aqueous phase [46].

The influence of the initial pH of the feed solutions on the La(III) loading capacity of the adsorbent MNP@TiO_2_-GA-H_3_PO_4_ was evaluated. Experiments were conducted within a pH range of 2 to 6, considering that at pH values above 7, lanthanides tend to precipitate as hydroxides, thereby reducing the concentration of dissolved ions in the solution. This behavior is supported by the speciation diagram presented in Figure 13 [46]. This figure shows that lanthanum ions are available in their free 3+ form from low pH values up to a range of approximately 6.5–7; beyond this interval, the predominant species change significantly, forming various hydroxylated species.

**Figure 14:**
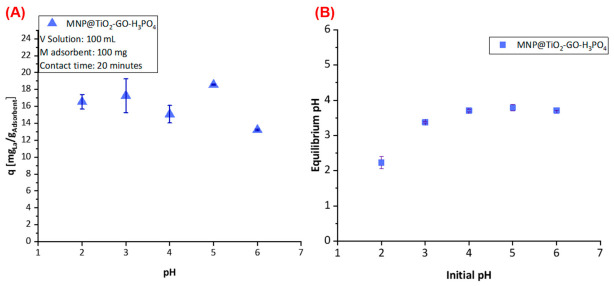
(**A**) Effect of initial pH on the adsorption of La(III) using MNP@TiO_2_-GA-H_3_PO_4_. Error bars represent the standard deviation (±SD). (**B**) Equilibrium pH vs initial pH.

The results obtained from the La(III) adsorption study are presented in Figure 14.

Figure 14A presents the loading capacity achieved for La(III) ions across the evaluated pH range. It can be observed that MNP@TiO_2_-GA-H_3_PO_4_ maintains high loading capacities throughout the entire pH range, reaching an experimental capacity of approximately 18.5 mg_La3+_\g_MNP_ at pH 5, which closely anticipates the fast kinetic responses. As previously indicated by the zeta potential study, the nanoparticle surface is negatively charged within this pH range, which promotes the electrostatic attraction of La^3+^ ions. Furthermore, the decline observed at pH 6 is likely due to the emergence of hydroxylated lanthanum species, as shown in the chemical speciation diagram.

On the other hand, Figure 14B displays the equilibrium pH of the resulting solutions as a function of the initial feed pH. An increase is observed at low pH values, reaching an equilibrium pH of 3.7 when the initial pH was 4. This shift indicates a slight release of protons from the functionalized surface into the liquid phase, suggesting the involvement of phosphate groups in the ion-exchange process during La(III) adsorption. This behavior has been reported for materials bearing organophosphorus groups, where metal–oxygen complexation occurs simultaneously with surface deprotonation [47]. Based on these results and aiming to maximize process efficiency, pH 5 was selected as the optimal condition for subsequent adsorption studies.

#### 4.3.2. Effect of Adsorbent Dosage on Adsorption

**Figure 15:**
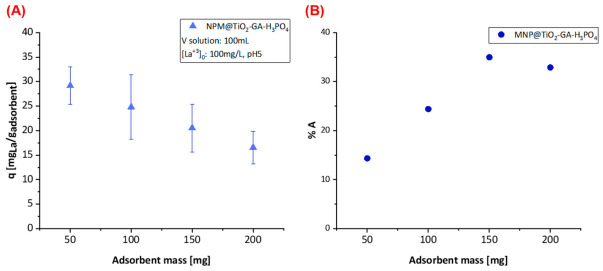
Effect of MNP@TiO_2_-GA-H_3_PO_4_ adsorbent mass on (**A**) loading capacity [mg/g] and (**B**) adsorption percentage.

The effect of adsorbent mass on La(III) adsorption using MNP@TiO_2_-GA-H_3_PO_4_ was evaluated. The study was conducted with a 100 ppm La(III) feed solution adjusted to pH 5, while the adsorbent dosage was varied between 50 and 200 mg (Figure 15).

Figure 15A shows that as the adsorbent mass increases, the loading capacity (q) decreases. This behavior is primarily attributed to a mass-loading effect; at a fixed lanthanum concentration, increasing the adsorbent mass results in a limited amount of ions being distributed across a larger quantity of material, thereby reducing the amount adsorbed per unit mass. Additionally, higher dosages may promote partial agglomeration of the nanoparticles, which reduces the effective specific surface area available for adsorption [48].

Figure 15B shows that the adsorption percentage (A%) increases with adsorbent mass, which is consistent with the greater availability of active sites for lanthanum retention. However, this rise in A% does not necessarily imply an increase in loading capacity (q). Since the adsorbent mass increases simultaneously, the total amount of adsorbed lanthanum is distributed over a larger mass of material, leading to a lower q value expressed in mg_La3+_/g_MNP_. An adsorbent dose of 100 mg was selected for subsequent experiments, as it represents an optimal balance between high loading capacity and a substantial adsorption percentage, while minimizing negative effects associated with particle agglomeration.

**Figure 16:**
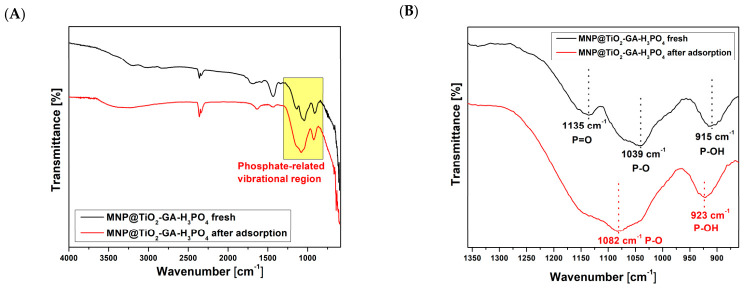
FT-IR spectra of the MNP@TiO_2_-GA-H_3_PO_4_ nanoadsorbent before and after La^3+^ ion adsorption: (**A**) full spectral range and (**B**) magnified view of the phosphate-related vibrational region between 1350 and 900 cm^−1^.

The FT-IR spectra of the adsorbent before and after lanthanum adsorption are presented in Figure 16 to provide evidence of its interaction with the phosphate groups of the nanoadsorbent.

To further elucidate the involvement of phosphate groups in REE adsorption, FT-IR spectra of MNP@TiO_2_-GA-H_3_PO_4_ were recorded before and after metal uptake. As illustrated in Figure 16A, the fresh or unloaded adsorbent exhibited characteristic phosphate-related bands at 1135, 1039, and 915 cm^−1^, which are assigned to P=O, P–O, and P–OH vibrations, respectively. Following adsorption, Figure 16B, the P–O stretching band significantly shifted to 1082 cm^−1^, while the P–OH band migrated to 923 cm^−1^. These pronounced shifts within the phosphate-related vibrational region reveal a distinct modification of the local chemical environment surrounding the functional groups, confirming their active participation in metal binding through the formation of REE–O–P coordination interactions [49,50].

### 4.4. Adsorption Kinetics of Lanthanum(III)

**Figure 17:**
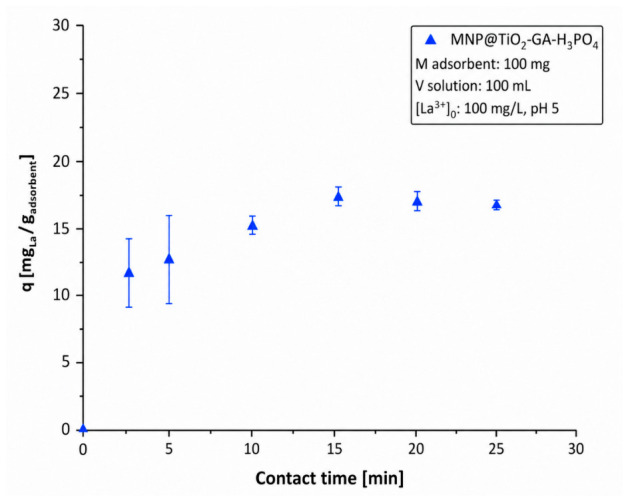
Adsorption kinetics of La(III) using MNP@TiO_2_-GA-H_3_PO_4_.

Kinetic experiments were conducted to study the evolution of adsorption over time and to determine the time required to reach the maximum loading capacity with the synthesized adsorbent. The obtained results are presented in Figure 17.

As shown in Figure 17, under the specified operating conditions, the maximum loading capacity is achieved after approximately 15 min of contact, reaching an experimental kinetic value of 18 mg_La3+_/g_MNP_.

Based on the experimental results, adsorption kinetic models were applied to evaluate the initial adsorption rate of La^3+^ and to determine kinetic parameters, such as the rate constant and theoretical equilibrium loading capacity. Furthermore, these models were used to identify the potential adsorption mechanisms controlling the process. In this study, pseudo-first-order and pseudo-second-order models were exclusively considered.

The pseudo-first-order kinetic model, also known as the Lagergren model, proposes that the adsorption rate is proportional to the number of available active sites on the adsorbent surface [51]. This model assumes that the adsorption process is primarily controlled by an external mass transfer mechanism—specifically, the diffusion of the adsorbate from the bulk aqueous solution to the solid–liquid interface. This model is mathematically expressed by Equation (3).

(3) $$q_{t}=q_{e}\left(1-e^{-k_{1}t}\right)$$

where *q_t_* is the loading capacity at time *t* (mg_La3+_/g_adsorbent_), *t* is the contact time (min), *k*_1_ is the adsorption rate constant (min^−1^), and *q_e_* is the equilibrium loading capacity (mg_La3+_/g_adsorbent_).

The pseudo-second-order model, also known as the Ho model, assumes that all adsorption sites on the adsorbent surface are homogeneous and that the adsorption process depends on both the number of available sites and the adsorbate concentration in the aqueous phase. This model can be mathematically expressed by Equation (4) [52].

(4) $$q_{t}=\frac{q_{e}^{2}k_{2}t}{1+q_{e}k_{2}t}$$

where *q_t_* and *q_e_* are the loading capacities at time *t* and at equilibrium, respectively, *t* is the time (min), and *k*_2_ is the rate constant (g_adsorbent_/mgLa_3+_⋅min). In this model, it is noteworthy that the term *k*_2_*q_e_*^2^ represents the initial adsorption rate (*v*_0_). When experimental data mathematically fit this model, it is conventionally associated with systems where the rate-limiting step matches pseudo-second-order kinetics. However, this numerical compliance should be treated as a macro-scale kinetic description rather than absolute proof of direct chemical sharing. As such, these findings serve as empirical indicators of the process rates, and any explicit molecular mechanism must be rigorously validated by advanced surface analysis in a future study [53].

**Figure 18:**
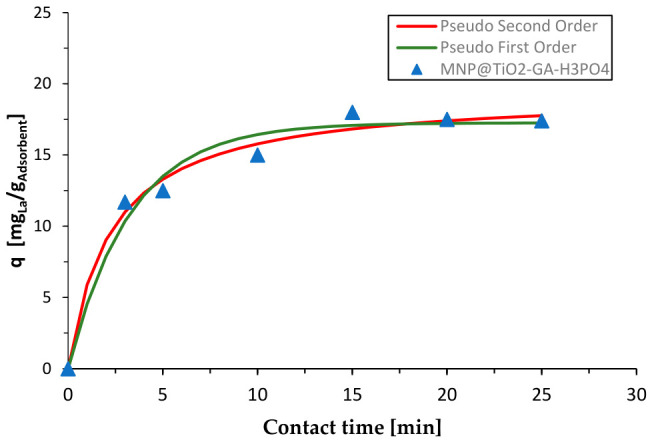
Fitting of pseudo-first-order and pseudo-second-order kinetic models to the experimental data for La(III) adsorption using MNP@TiO_2_-GA-H_3_PO_4_.

The application of the pseudo-first-order and pseudo-second-order kinetic models is presented in Figure 18, while their corresponding fitting parameters are summarized in Table 4.

Figure 18 shows that the initial adsorption rate is rapid, with the maximum loading capacity being reached within approximately 15 min of contact. This result indicates a fast initial interaction between the adsorbate and the active sites on the surface of the nanoadsorbents.

Table 4 presents the kinetic parameters obtained for both models, along with the correlation coefficients (*R^2^*) and the chi-square (*χ^2^*) values, which allow for the evaluation of the goodness-of-fit in non-linear models and the comparison of the kinetic behavior of each system.

According to the statistical parameters presented in Table 4, the pseudo-second-order model exhibited the best fit, with correlation coefficients closer to unity and lower chi-square values, indicating a superior representation of the experimentally observed kinetic behavior. This fitting model suggests that the adsorption rate depends primarily on the availability of active sites on the material’s surface.

### 4.5. Adsorption Equilibrium of Lanthanum(III)

To evaluate the adsorption equilibrium behavior of lanthanum using MNP@TiO_2_-GA-H_3_PO_4_, experiments were conducted using feed solutions with initial concentrations of 30, 40, 60, 80, and 100 mg/L. For each experimental condition, 100 mg of adsorbent and 100 mL of solution were kept in contact via mechanical stirring for 15 min at room temperature.

Following the contact time, the solutions were separated by magnetic decantation, and the final equilibrium concentration of lanthanum (*C_e_*) was determined. The results can be interpreted by fitting theoretical adsorption models, which provide information regarding the nature of the adsorption process, specifically whether it occurs via monolayer or multilayer formation. Generally, monolayer adsorption is associated with a chemisorption process, whereas multilayer adsorption corresponds to physisorption or mixed-cooperative models.

The adsorption models applied to the experimental data in this study were the Langmuir, Freundlich, and the Sips mixed model. These are theoretical models that indicate the equilibrium behavior trends of the synthesized MNPs. The Langmuir model is an ideal theoretical framework that assumes adsorption occurs on a homogeneous surface where all active sites are energetically equivalent. Furthermore, it considers that each active site can be occupied by only a single adsorbate molecule, resulting in the formation of a monolayer. This model assumes no interactions between adsorbed species nor migration across the adsorbent surface [54] and is mathematically expressed by Equation (5).

(5) $$q_{e}=\frac{q_{m}K_{L}C_{e}}{1+K_{L}C_{e}}$$

where *q_e_* is the loading capacity at equilibrium (mg_metal_/g_adsorbent_), *q_m_* is the maximum loading capacity (mg_metal_/g_adsorbent_), *K_L_* is the Langmuir constant (L/mg) related to the affinity between the adsorbent and the adsorbate, and *C_e_* is the equilibrium concentration of the adsorbate (mg/L).

The Freundlich model is a non-ideal, reversible model used to describe adsorption processes on heterogeneous surfaces. Unlike the Langmuir model, it assumes that adsorption occurs via multilayer formation. Furthermore, it considers that the active adsorption sites possess varying energy levels, which are preferentially occupied by molecules with the highest affinity during the initial stages [55]. The model is described by Equation (6).

(6) $$q_{e}=K_{F}C_{e}^{\frac{1}{n}}$$

where *q_e_* is the loading capacity achieved at equilibrium (mg_metal_/g_adsorbent_), *K_F_* is the Freundlich constant [(mg/g)(L/mg)^1/*n*^], which relates to the adsorption capacity of the adsorbent, 1/*n* is related to the adsorption intensity, and *C_e_* is the equilibrium concentration of the adsorbate (mg/L).

**Figure 19:**
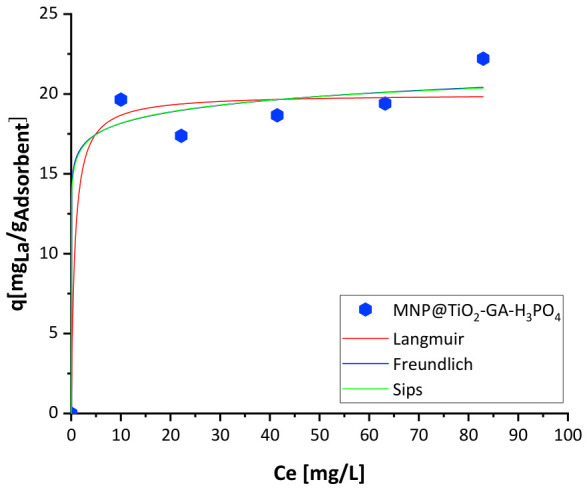
Adsorption equilibrium results for La(III) using MNP@TiO_2_-GA-H_3_PO_4_ and the fitting of Langmuir, Freundlich, and Sips models.

The Sips model, also known as the Langmuir–Freundlich model, is a combination of the previously described models. This hybrid approach enables the representation of both monolayer and multilayer adsorption processes, making it useful for describing partially heterogeneous surfaces. The model assumes that at low adsorbate concentrations, the behavior approximates the Freundlich model, whereas at high concentrations, it tends toward Langmuir-like behavior [56]. The mathematical expression of the model is described in Equation (7).

(7) $$q_{e}=\frac{q_{m}K_{LF}C_{e}^{\frac{1}{n}}}{1+K_{LF}C_{e}^{\frac{1}{n}}}$$

where *q_e_* and *q_m_* are the equilibrium and maximum loading capacities (mg_metal_/g_adsorbent_), respectively; *K_LF_* is the Sips constant (L^1/*n*^⋅mg^−1/*n*^); 1/*n* is a factor related to the adsorption intensity; and *C_e_* is the equilibrium adsorbate concentration (mg/L). When *n* is equal to unity, the equation is equivalent to the Langmuir model. The application of these equilibrium models to the experimental data is presented in Figure 19, and their corresponding fitting parameters are summarized in Table 5.

Figure 19 presents the experimental results for the loading capacities achieved by MNP@TiO_2_-GA-H_3_PO_4_ within the studied concentration ranges. Table 5 summarizes the equilibrium parameters obtained by fitting the adsorption models, along with their respective correlation coefficients and error values, which allow for the identification of the model that most accurately describes the experimental behavior of the system.

Among the models evaluated for La(III) adsorption using MNP@TiO_2_-GA-H_3_PO_4_, the Freundlich model exhibited the best experimental fit, yielding the highest correlation coefficient (*R*^2^ = 0.97) and the lowest chi-square (*χ*^2^ = 2.364) value. This model suggests an adsorption process occurring on a heterogeneous surface, which is consistent with the presence of multiple functionalized active sites on the chemically modified and coated magnetite nanoparticles. The parameter value of *n* = 18.08 indicates favorable adsorption, as values of *n* > 1 are associated with a high affinity between the adsorbate and the adsorbent.

To contextualize the performance of the MNP@TiO_2_-GA-H_3_PO_4_ nanoadsorbent, a comparative analysis of maximum loading capacities for various lanthanides was conducted using other adsorbent materials reported in the literature; their values are summarized in Table 6. The adsorption capacity of the synthesized material aligns with the mid-range performance of currently available adsorbents.

### 4.6. Desorption, Regeneration, and Reusability of the Adsorbent

To evaluate the regeneration capacity and reusability of the adsorbent, preliminary desorption experiments were conducted using lanthanum (III)-loaded functionalized nanoparticles (MNP@TiO_2_-GA-H_3_PO_4_). These experiments were designed based on the premise that the titanium dioxide coating, particularly in its anatase phase, provides the nanoparticles with enhanced resistance in acidic media, thereby allowing them to withstand harsh pH conditions [21].

**Figure 20:**
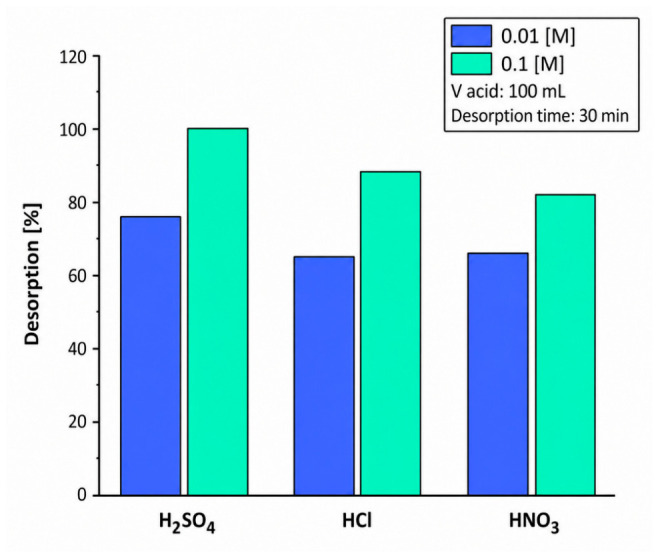
Desorption profiles of La(III) from loaded MNP@TiO_2_-GA-H_3_PO_4_.

Desorption experiments were conducted using three mineral acids—hydrochloric acid (HCl), sulfuric acid (H_2_SO_4_), and nitric acid (HNO_3_)—at concentrations of 0.1 M and 0.01 M. This study aimed to evaluate the relative efficacy of each eluent in recovering the adsorbed lanthanum ions and to optimize preliminary desorption parameters. As shown in Figure 20, desorption efficiency was calculated relative to the initial mass (mg) of La(III) loaded onto the MNP@TiO_2_-GA-H_3_PO_4_ nanoparticles, defined as the theoretical maximum (100%) recovery.

The results presented in Figure 20 demonstrate that the desorption percentage increases with acid concentration in all cases. Sulfuric acid (H_2_SO_4_) exhibited the highest performance, achieving complete desorption (100%) at a concentration of 0.1 M. Based on these findings, H_2_SO_4_ was selected as the eluent for subsequent reusability studies [61].

To evaluate the stability and reusability of the MNP@TiO_2_-GA-H_3_PO_4_ material, five consecutive adsorption–desorption cycles were conducted using lanthanum solutions under optimized conditions (pH 5, 15 min contact time, and an initial concentration of 100 ppm). For the initial cycle, the 1 g mass of lanthanum loaded onto the adsorbent was established as the 100% baseline. Desorption efficiency was subsequently calculated relative to this value, enabling the assessment of the material’s regeneration performance by tracking mass recovery throughout consecutive adsorption–desorption cycles.

**Figure 21:**
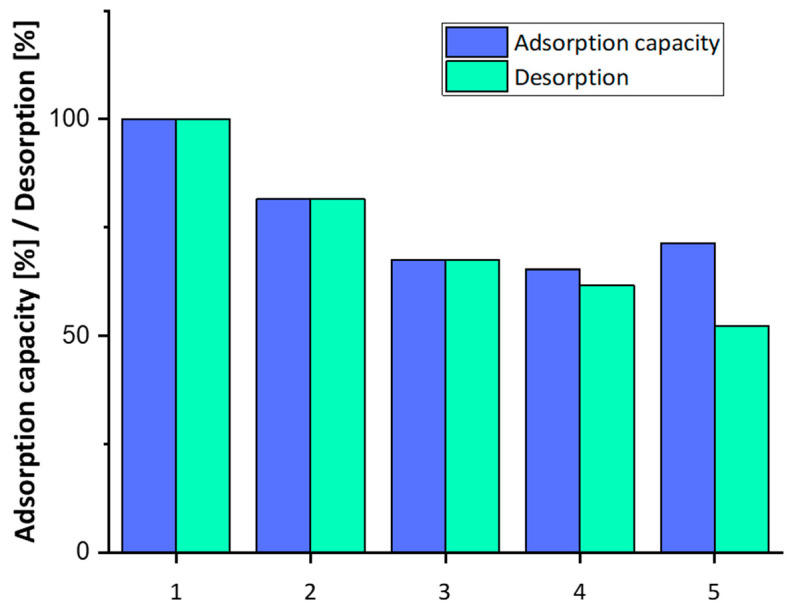
Reusability study of MNP@TiO_2_-GA-H_3_PO_4_ for the adsorption and desorption of lanthanum (III).

The results of the regeneration study are presented in Figure 21. Adsorption capacity decreased to 75% after five consecutive cycles, while desorption efficiency declined to approximately 58–60%.

These findings demonstrate the material’s chemical stability and the robust, reversible interaction between the phosphate groups on the functionalized surface and the lanthanum ions. The observed progressive decrease in desorption efficiency, which drops to approximately 58–60% by the fifth cycle, is driven by three primary mechanisms. First, continuous mass loss during successive handling steps—from an initial 0.1017 g to 0.076 g—directly accounts for the 25% reduction in overall adsorption capacity (retaining 75% of its baseline value). Second, irreversible site occupation takes place as a minor fraction of the trivalent REE ions forms highly stable coordination complexes with the high-density phosphate groups, resisting complete cleavage by the 0.1 M H_2_SO_4_ eluent during the 15-min desorption step. Third, repeated magnetic separation and mechanical handling induce aggregate compaction of the 125.6 nm hydrodynamic clusters. This structural reorganization physically entraps a minority of ions within internal cavities, thereby hindering eluent mass transport and limiting full desorption.

Collectively, these findings confirm that the MNP@TiO_2_-GA-H_3_PO_4_ nanomaterial exhibits remarkable reusability, maintaining high lanthanum adsorption efficiency for at least four consecutive cycles without a substantial decline in performance. This underscores its potential suitability for sustainable rare earth element (REE) recovery processes.

### 4.7. Metallic Content from LCD Screen Leachates

Three types of LCD screen waste samples were considered for this study—specifically S-1, S-2, and S-3—which were subjected to a sequential pretreatment involving dismantling, crushing, and grinding. The resulting material was analyzed by SEM-EDX, confirming the aluminosilicate nature of the substrate, as evidenced by the presence of silicon and aluminum. The identification of Cr, Fe, and Ni suggests the inclusion of metallic alloys from reinforcements or coatings, while the detection of Cu is attributed to internal wiring and connectors.

Aqueous solutions containing various metallic components were subsequently obtained by leaching the ground LCD samples. Specifically, 25 g of the ground material was leached with 140 mL of aqua regia—a mixture of concentrated nitric acid and hydrochloric acid in a 1:3 volumetric ratio, respectively. The resulting solution was kept under stirring for 24 h. Subsequently, the mixture was filtered, and the pH was adjusted to 5.0 via the dropwise addition of 2.0 M NaOH to induce the precipitation of bulk transition metal impurities. The resulting dense metal-hydroxide sludges were then separated via vacuum filtration. Mass-balance tracking revealed that this pre-neutralization step achieved high removal efficiencies, with precipitation rates reaching 96.3% for Fe and 85.5% for Cu, while target REE co-precipitation remained negligible. The final supernatant was diluted to a volume of 250 mL and utilized for subsequent elemental analysis.

Atomic absorption spectroscopy and ICP-MS analyses revealed that calcium (Ca) is the most abundant element in the leachate, with concentrations reaching up to 97.82 ppm. This prevalence is consistent with the composition of LCD glass, where calcium is essential for the material’s mechanical integrity and structural stability. The chemical compositions of the various leachate samples are summarized in Table 7.

ICP-MS was employed to complement the elemental analysis of the LCD screen leachates, enabling the detection of rare earth elements (REEs) present at trace levels. This technique allowed for a more precise characterization of the leachate composition following pH adjustment, providing critical insights into the elemental distribution and the efficacy of the leaching process. The concentrations determined after pH stabilization are summarized in Table 8.

These results indicated that while Gd was detected in all samples, Y, Ce, Pr, Nd, and Sm were present in only two.

### 4.8. Adsorption Study of Metallic Content from Leachates Using MNP@TiO_2_-GA-H_3_PO_4_

Preliminary analyses of the LCD leachates identified a diverse range of elements, including light rare earth elements (REEs: Y, Ce, Nd, Sm, and Gd) alongside interfering transition metals such as Fe, Ni, Cu, and Cr, which may compete for the adsorbent’s active sites. Given this complex matrix, adsorption assays were conducted using the optimized parameters previously established for synthetic solutions: pH 5, an adsorbent dose of 100 mg per 100 mL of leachate, and a 15-min contact time. These experiments were designed to evaluate the operational performance of the MNP@TiO_2_-GA-H_3_PO_4_ nanoadsorbent within a challenging multi-elemental system.

The adsorption capacities (*q_e_*, mg/g), corresponding removal efficiencies (*R%*) and selectivity coefficients achieved for each element identified in the S-3 leachate are summarized in Table 9.

To mathematically standardize the competitive affinity of the MNP@TiO_2_-GA-H_3_PO_4_ nanoadsorbent across widely differing initial concentration scales, the normalized distribution coefficient (*K_d_*) was determined (Table 9). When assessing these metrics under realistic multi-element conditions, a defining process parameters configuration must be explicitly highlighted: the real electronic waste leachate utilized for this validation represents a preconditioned hydrometallurgical supernatant. Prior to reaching the adsorption stage, the raw crude aqua regia leachate underwent a mandatory neutralization step adjusting the medium to pH 5.0. This chemical preconditioning successfully forced the partial precipitation of massive baseline concentration quantities of iron and copper. Such upstream separation is operationally vital; if left unconditioned, the extreme mass-action effects of macro-concentrations of transition metals would rapidly crowd out and completely blind the active organophosphorus surface receptor sites.

The thermodynamic data trends in Table 9 demonstrate that despite this preconditioning, residual base metal concentrations still exert high competitive pressure. Nickel displays a complete lack of affinity toward the functionalized surface (*K_d_* = 0.0 mL/g), and residual iron uptake is strongly minimized (*K_d_* = 7.6 mL/g), which confirms high programmatic tolerance toward these matrix components. However, copper (*K_d_* = 798.6 mL/g) and chromium (*K_d_* = 347.7 mL/g) remain highly aggressive competitive interceptors due to their elevated lingering ppm-scale concentrations in the preconditioned liquid phase. Regarding the strategic elements, the nanoadsorbent confirms its distinct technical potential by selectively isolating trace rare earth ions from the multi-element blend, achieving high normalized distribution coefficients prominently topped by samarium (*K_d_* = 1000.0 mL/g) and gadolinium (*K_d_* = 308.9 mL/g). These values robustly validate that the material maintains an elevated localized chemical affinity for trivalent lanthanides even when submerged under a highly challenging, non-ideal urban mining matrix. To further quantify the relative separation performance among the target lanthanides, the cross-selectivity factors (S_M1/M2_) were calculated and summarized in Table 10.

To gain a deeper insight into the competitive thermodynamic behavior specifically within the rare earth fraction, the cross-selectivity factors (S_M1/M2_) were evaluated by plotting the ratio of their respective distribution coefficients (*K_d_*) in a matrix format (Table 10). In this multi-component framework, values greater than 1.0 indicate a distinct chemical preference for the column element (M_1_) over the row element (M_2_).

The numerical data of Table 10 reveals several prominent selectivity trends:

- Absolute Dominance of Samarium: Samarium (Sm) exhibits a remarkably high separation factor against all other lanthanides, with values peaking at 15.8 against praseodymium (Pr) and 15.3 against yttrium (Y). This behavior confirms that the dual glycolic/phosphoric functional groups possess an enhanced structural configuration that preferentially complexes medium REEs under highly competitive conditions.
- Prominence of Gadolinium: Gadolinium (Gd) displays a strong secondary affinity, consistently outperforming light rare earths with separation factors such as 4.9 against Pr and 4.2 against neodymium (Nd).
- Co-adsorption Behaviors: In contrast, the lighter REEs (Y, Ce, Pr, and Nd) exhibit cross-selectivity factors hovering close to unity, ranging strictly from 0.7 to 1.3. This sub-system behavior indicates a highly uniform co-adsorption pattern. This trend is structurally consistent with the classic lanthanide contraction effect, where light rare earths possess highly similar ionic radii and identical trivalent charges, leading to equivalent mass-action responses on the heterogeneous surface active sites.

In summary, the practical validation of the MNP@TiO_2_-GA-H_3_PO_4_ nanoadsorbent in real multi-element matrices demonstrates its viability as a high-performance hydrometallurgical asset. While the presence of aggressive residual transition metals like copper and chromium highlights the absolute necessity of maintaining a highly controlled upstream pH 5.0 preconditioning barrier, the material’s capacity to actively scavenge and partition strategic REEs present at ultra-low trace levels (µg/L) is remarkable. By coupling the high chemical stability of the anatase phase shell with the rapid, magnetically assisted recovery of the underlying core, this core–shell architecture successfully transitions from a laboratory proof-of-concept into a highly sustainable technology for urban mining and the circular economy.

## 5. Discussion

The development of the phosphate-functionalized magnetic nanoadsorbent (Fe_3_O_4_@TiO_2_-GA-H_3_PO_4_) represents a significant advancement in the field of urban mining, specifically for the selective recovery of rare earth elements (REEs) from complex electronic waste.

The core–shell architecture was designed to address two critical limitations in current adsorption technologies: chemical instability and separation difficulty.

- Chemical Stability: The formation of an anatase-phase TiO_2_ shell, with a calculated thickness of 2.4 ± 0.8 nm, provided a refractory protective coating that allowed the magnetite core to withstand the acidic conditions required for REE processing.
- Magnetic Recovery: Despite the non-magnetic layers introduced by the coating and functional groups, the material retained superparamagnetic behavior with a saturation magnetization of 1.9 emu/g. This supports the hypothesis that magnetically assisted chemical separation (MACS) can replace energy-intensive centrifugation in industrial workflows.

Furthermore, the scientific novelty of the MNP@TiO_2_-GA-H_3_PO_4_ nanoadsorbent rests on the structure–performance synergy of its 2.4 nm anatase shell and a dual glycolic/phosphoric acid functionalization pathway. Although the 16.1 nm primary units form 125.6 nm hydrodynamic clusters, this open porosity eliminates mass transport resistance, achieving full adsorption equilibrium in just 15 min. Operationally, dense phosphate grafting provides a stable −33 mV surface charge preventing coagulation, while a polydisperse “magnetic sweeping” mechanism overcomes the low 1.9 emu/g magnetization, reducing clearing times to 0.6 min. This multi-scale optimization efficiently scavenges strategic rare earths at ultra-low trace levels (µg/L) from complex real LCD leachates.

The adsorption performance aligns with and, in some aspects, improves upon traditional solvent extraction (SX) and previous adsorbents:

- The superior fit of the pseudo-second-order model (*R^2^* = 0.989) indicates a highly rapid macroscopic uptake controlled by the availability of surface active sites. While this numerical trend phenomenologically resembles the behavior of successful liquid organophosphorus extractants used in the mining industry, it is treated here strictly as an empirical parameter fit for process scaling rather than a definitive molecular-level proof of coordination geometry. Similarly, the alignment with the Freundlich model parameters (*R^2^* = 0.97) macroscopically describes adsorption on a functionally heterogeneous surface. In the absence of post-adsorption characterization (such as XPS or localized EDS mapping), these models are not used to draw definitive mechanistic conclusions, which remain a subject for future spectroscopic validation.

The successful application of the nanoadsorbent on real LCD screen leachates demonstrates its viability beyond idealized laboratory conditions.

- Selectivity Challenges: While the material effectively captured REEs (Gd, Y, Ce, Pr, Nd, and Sm) at trace levels (μg/L), the high adsorption of copper and chromium identifies these as primary competitors in the multi-elemental matrix of e-waste.
- Circular Economy: The ability to regenerate the material using H_3_PO_4_ and maintain 75% of its capacity after five cycles underscores its potential for a sustainable, closed-loop recycling system. This is critical given that only 22.3% of global e-waste is currently recycled formally.

To transition this technology from laboratory “proof-of-concept” to industrial application, future studies should focus on:

- Interferent Mitigation: Developing pre-treatment stages to specifically remove copper and chromium prior to REE adsorption to prevent active site saturation.
- Scaling: Investigating the performance of the nanoadsorbent in continuous flow systems or large-scale magnetically assisted reactors.
- Mass Loss Prevention: Addressing the decline in desorption efficiency (which dropped to 58–60% by the fifth cycle) by optimizing the washing and magnetic recovery steps to minimize loss of the nanostructured material.

## 6. Conclusions

Regarding the development and performance of the MNP@TiO_2_-GA-H_3_PO_4_ nanoadsorbent for the recovery of rare earth elements, the following conclusions can be drawn:

- A core–shell structured material, consisting of a magnetite (Fe_3_O_4_) core and an anatase-phase titanium dioxide shell, was successfully synthesized. The TiO_2_ coating provided chemical stability in acidic media, while the core retained its superparamagnetic properties, enabling efficient magnetic separation from the solution. The final functionalization with organophosphorus groups was verified by a shift in zeta potential and the emergence of phosphate group vibration bands in the FT-IR spectrum.
- Optimal adsorption was achieved at pH 5, evaluating an experimental kinetic capacity of 18.5 mg/g at 15 min, while fitting a theoretical capacity for lanthanum at 19.4 ± 0.8 mg/g through kinetic modeling. The experimental data showed a high mathematical correlation with the pseudo-second-order kinetic and Freundlich equilibrium equations. This provides a robust empirical framework for understanding macroscopic process rates and surface heterogeneity, while leaving the explicit chemical binding mechanisms open for future post-adsorption surface characterization.
- The adsorbent demonstrated high chemical stability, retaining 75% of its initial adsorption capacity after five consecutive adsorption–desorption cycles. Sulfuric acid (H_2_SO_4_ 0.1 M) proved to be the most effective eluent, achieving complete (100%) desorption of the adsorbed metal during the initial cycles. While adsorption remained robust, desorption efficiency declined to approximately 58–60% by the fifth cycle, likely due to the partial saturation of active sites or minor adsorbent mass loss during processing.
- Analytical results confirmed the presence of rare earth elements (REEs)—specifically Gd, Y, Ce, Pr, Nd, and Sm—within actual LCD leachate samples at trace levels (μg/L). The operational validation demonstrated that an upstream pH 5.0 pre-neutralization and filtration step is essential to mitigate severe mass-action effects from macro-concentrations of iron and copper. Following this pretreatment, the MNP@TiO_2_-GA-H_3_PO_4_ nanoadsorbent effectively recovered these REEs even at trace concentrations. While the pre-treated matrix exhibited no further affinity for Ni and minimal affinity for Fe, it was determined that residual Cu and Cr act as the principal interferents, directly competing for active sites within the complex multi-elemental matrix.
- The technical advance of this work relies on the successful integration of the protective TiO_2_ shell with the dual-acid functional network, systematically validated in a real, highly complex LCD leachate matrix. Despite the low μg/L trace-level concentrations of target lanthanides and the overwhelming presence of transition-metal interferents, the material demonstrated robust selective partitioning. This establishes the practical viability of this specific material configuration for realistic urban mining and circular economy applications.

## Figures and Tables

**Table 1:** Magnetic parameters of MNPs and MNP@TiO_2_.

Nanoparticle	M_s_ (emu/g)	M_r_ (emu/g)	$M_{r}/M_{s}$
MNP	60	0	0
MNP@TiO_2_	4.7	0.2	0.043

**Table 2:** Comparison of magnetic parameters for the coated and functionalized nanoparticles.

Nanoparticles	M_s_ (emu/g)	M_r_ (emu/g)	$M_{r}/M_{s}$
MNP@TiO_2_	4.7	0.2	0.043
MNP@TiO_2_-GA-H_3_PO_4_	1.9	0.15	0.079

**Table 3:** Evaluation of the practical magnetic separation performance and mass recovery efficiency of MNP@TiO_2_-GA-H_3_PO_4_ nanoparticles over five consecutive handling cycles (initial mass: 0.1017 g).

Cycle	Separation Time [min]	Final Mass [g]	Mass Loss [g] *	Magnetic Recovery [%]
1	2	0.095	0.0067	93.4
2	1.8	0.089	0.0062	93.5
3	1.3	0.089	0.0003	99.7
4	0.8	0.081	0.0075	91.5
5	0.6	0.076	0.0055	93.2

* Mass loss calculated relative to the remaining mass of the previous cycle.

**Table 4:** Parameters obtained from the fitting of kinetic models.

**Pseudo-First-Order**	***q_e_*** ** [mg_La_·g_MNP_^−1^]**	***k*** **_1_ [min^−1^]**	***R*** **^2^**	***χ*** **^2^**
MNP@TiO_2_-GA-H_3_PO_4_	17.25	0.31	0.976	0.4326
**Pseudo-Second-Order**	***q_e_*** ** [mg_La_·g_MNP_^−1^]**	***k*** **_2_** ** [g_MNP·_mg_La_^−1^·min^−1^]**	***R*** **^2^**	***χ*** **^2^**
MNP@TiO_2_-GA-H_3_PO_4_	19.39	0.022	0.986	0.2219

**Table 5:** Equilibrium parameters obtained from the fitting of Langmuir, Freundlich, and Sips models.

**Langmuir**	***q_m_*** ** [mg_La_ g_MNP_^−1^]**	***K_L_*** ** [L mol^−1^]**	***R* ^2^**		***χ* ^2^**
MNP@TiO_2_-GA-H_3_PO_4_	20	1.41	0.96		2.936
**Freundlich**	***n***	***K_F_* [L^*n*^ mg^1−*n*^ g^−1^]**	***R* ^2^**		***χ* ^2^**
MNP@TiO_2_-GA-H_3_PO_4_	18.08	15.98	0.97		2.364
**Sips**	***q_m_* [mg_La_ g_MNP_^−1^]**	***K_LF_* [L^1/*n*^ mg^−1/*n*^]**	***n***	***R* ^2^**	***χ* ^2^**
MNP@TiO_2_-GA-H_3_PO_4_	42.03	0.006	10	0.96	3.2

**Table 6:** Maximum loading capacities for selected lanthanides using different adsorbent materials.

Adsorbent Material	Target REE	Adsorption Capacity (*q_max_*, mg/g)	Equilibrium Time (*t_eq_*)	Operating pH	Real Samples Tested	Reference
MNP@TiO_2_-GA-H_3_PO_4_	La(III)/Multi-REE	19.4 ± 0.8	15 min	5.0	Yes (Real LCD screen leachates)	This work
Walnut shell (WS, bioadsorbent)	La(III)	71	12 h (720 min)	4.0	No	[57]
Durian-derived activated carbon	La(III)	~71	120–240 min	5.0–6.0	No	[57]
Modified alginate/goethite (GSA-2)	La(III)	77.8	120–240 min	5.0–6.0	No	[58]
ACDs (activated carbon from date seeds)	La(III)/Ce(III)/Sm(III)	18.2/24.6/27.8	180 min	5.0–5.7	No	[59]
Nitrolite (resin)	La(III)	4.8	180 min (3 h)	9.0	No	[60]

**Table 7:** Chemical characterization of LCD screen leachates.

Sample	Element			
	Fe [ppm]	Cu [ppm]	Ni [ppm]	Ca [ppm]
S-1	0.304	0.141	20	54.14
S-2	2.922	1.071	37	81.76
S-3	1.864	0.679	77.08	97.82

**Table 8:** Concentration of rare earth elements in LCD screen leaching solutions determined by ICP-MS.

**Sample**	**Sc [μg/L]**	**Y** **[μg/L]**	**La [μg/L]**	**Ce [μg/L]**	**Pr [μg/L]**	**Nd [μg/L]**	**Eu [μg/L]**	**Sm** **[μg/L]**
S-1	<0.5	<0.5	<0.5	<0.5	<0.5	<0.5	<0.5	<0.5
S-2	<0.5	1.4	<0.5	5.3	2.2	7.7	<0.5	1.1
S-3	<0.5	1.9	<0.5	42.0	1.6	16.9	<0.5	1.1
**Sample**	**Gd** **[μg/L]**	**Tb [μg/L]**	**Dy [μg/L]**	**Ho [μg/L]**	**Er [μg/L]**	**Tm [μg/L]**	**Yb [μg/L]**	**Lu** **[μg/L]**
S-1	2.3	<0.5	<0.5	<0.5	<0.5	<0.5	<0.5	<0.5
S-2	5.2	<0.5	<0.5	<0.5	<0.5	<0.5	<0.5	<0.5
S-3	4.5	<0.5	<0.5	<0.5	<0.5	<0.5	<0.5	<0.5

**Table 9:** Initial vs. equilibrium concentrations, capacities, and distribution coefficients (*K_d_*) for metallic elements from S-3 leachate using the MNP@TiO_2_-GA-H_3_PO_4_ nanocomposite (V = 100 mL, M = 100 mg, pH = 5.0, t = 15 min).

Element Type	Element	Initial Concentration (*C_i_*)	Equilibrium Concentration (*C_e_*)	Adsorption Capacity (*q_e_*)	Removal Efficiency (*R%*)	Distribution Coefficient (*K_d_*, mL/g)	Selectivity Coefficient
Base Metals	Fe	1.864 mg/L	1.850 mg/L	0.014 mg/g	0.75%	7.6	S_Cu/Fe_: 105.0
	Cu	0.679 mg/L	0.378 mg/L	0.301 mg/g	44.4%	798.6	S_Cu/Cu_: 1
	Ni	77.080 mg/L	77.080 mg/L	0.000 mg/g	0.00%	0.0	S_Cu/Ni_: ∞
	Cr	21.410 mg/L	15.886 mg/L	5.524 mg/g	25.80%	347.7	S_Cu/Cr_: 2.3
Rare Earths	Y	1.900 μg/L	1.783 μg/L	1.17 × 10^−4^ μg/g	6.15%	65.5	S_Sm/Y_: 15.3
	Ce	42.000 μg/L	38.186 μg/L	3.81 × 10^−4^ μg/g	9.08%	99.9	S_Sm/Ce_: 10.0
	Pr	1.600 μg/L	1.505 μg/L	9.50 × 10^−5^ μg/g	5.94%	63.2	S_Sm/Pr_: 15.8
	Nd	16.900 μg/L	15.732 μg/L	1.17 × 10^−3^ μg/g	6.91%	74.2	S_Sm/Nd_: 13.5
	Sm	1.100 μg/L	0.550 μg/L	5.50 × 10^−4^ μg/g	50.00%	1000.0	S_Sm/Sm_: 1
	Gd	4.500 μg/L	3.438 μg/L	1.06 × 10^−3^ μg/g	23.60%	308.9	S_Sm/Gd_: 3.2

**Table 10:** Cross-selectivity factors (S_M1/M2_) between rare earth elements (REEs) during multicomponent adsorption from the preconditioned real S-3 LCD screen leachate using MNP@TiO_2_-GA-H_3_PO_4_ nanoparticles (V = 100 mL, M = 100 mg, pH 5.0, t = 15 min). The selectivity factor is defined as the ratio of their respective distribution coefficients: S_M1/M2_ = *K_d_*(M_1_)/*K_d_*(M_2_), where M_1_ represents the column element and M_2_ represents the row element.

S_M1/M2_		M_1_					
		Y	Ce	Pr	Nd	Sm	Gd
M_2_	Y	1.0	1.5	1.0	1.1	15.3	4.7
	Ce	0.7	1.0	0.6	0.7	10.0	3.1
	Pr	1.0	1.6	1.0	1.2	15.8	4.9
	Nd	0.9	1.3	0.9	1.0	13.5	4.2
	Sm	0.1	0.1	0.1	0.1	1.0	0.3
	Gd	0.2	0.3	0.2	0.2	3.2	1.0

## Data Availability

The original contributions presented in this study are included in the article. Further inquiries can be directed to the corresponding author.

## Abbreviations

The following abbreviations are used in this manuscript:

REE	Rare Earth Element
WEEE	Waste electrical and electronic equipment
MNP	Magnetite nanoparticle
MNP@TiO_2_	Magnetite@titanium dioxide core–shell nanoparticles
MNP@TiO_2_-GA	Magnetite@titanium dioxide core–shell nanoparticles functionalized with Glycolic Acid
MNP@TiO_2_-GA-H_3_PO_4_	Magnetite@titanium dioxide core–shell nanoparticles functionalized with Glycolic acid and phosphoric acid
